# Genome-wide identification of disease-causing copy number variations in 450 individuals with anorectal malformations

**DOI:** 10.1038/s41431-022-01216-5

**Published:** 2022-11-01

**Authors:** Julia Fabian, Gabriel C. Dworschak, Lea Waffenschmidt, Luca Schierbaum, Charlotte Bendixen, Stefanie Heilmann-Heimbach, Sugirthan Sivalingam, Andreas Buness, Nicole Schwarzer, Thomas M. Boemers, Eberhard Schmiedeke, Jörg Neser, Johannes Leonhardt, Ferdinand Kosch, Sandra Weih, Helen Maya Gielen, Stuart Hosie, Carmen Kabs, Markus Palta, Stefanie Märzheuser, Lena Marie Bode, Martin Lacher, Frank-Mattias Schäfer, Maximilian Stehr, Christian Knorr, Benno Ure, Katharina Kleine, Udo Rolle, Marcin Zaniew, Grote Phillip, Nadine Zwink, Ekkehart Jenetzky, Heiko Reutter, Alina C. Hilger

**Affiliations:** 1 Institute of Human Genetics, Medical Faculty of the University Bonn & University Hospital Bonn, Bonn, Germany; 2 Department of Neuropediatrics, University Hospital Bonn, Bonn, Germany; 3 Institute of Anatomy, Medical Faculty, University of Bonn, Bonn, Germany; 4 Unit of Pediatric Surgery, Department of General, Visceral, Vascular and Thoracic Surgery, University Hospital Bonn, Bonn, Germany; 5 Institute for Medical Biometry, Informatics and Epidemiology, Medical Faculty, University of Bonn, Bonn, Germany; 6 Institute for Genomic Statistics and Bioinformatics, Medical Faculty, University of Bonn, Bonn, Germany; 7 Core Unit for Bioinformatics Data Analysis, Medical Faculty, University of Bonn, Bonn, Germany; 8 SoMA, The German Patient Support Organization for Anorectal Malformations and Hirschsprung Disease, Munich, Germany; 9 Department of Pediatric Surgery and Pediatric Urology, Children’s Hospital of Cologne Amsterdamer Strasse, Cologne, Germany; 10 Clinic for Pediatric Surgery and Pediatric Urology, Klinikum Bremen Mitte, Bremen, Germany; 11 Department of Pediatric Surgery, General Hospital, Chemnitz, Germany; 12 Department of Pediatric Surgery, Children’s Hospital Braunschweig, Braunschweig, Germany; 13 Department of Pediatric Surgery, Städtisches Klinikum Karlsruhe, Karlsruhe, Germany; 14 Department of Pediatric Surgery, Medical Center, Faculty of Medicine, University of Freiburg, Freiburg, Germany; 15 Department of Pediatric Surgery, Asklepios Klinik Nord Heidberg, Hamburg, Deutschland; 16 Muenchen Klinik gGmbH, Muenchen, Klinik Schwabing, Technische Universitaet Muenchen, Munich, Germany; 17 Department of Pediatric Surgery, Evangelisches Krankenhaus Hamm, Hamm, Germany; 18 Department of Pediatric Surgery, Rostock University Medical Center, Rostock, Germany; 19 Department of Pediatric Surgery, University of Leipzig, Leipzig, Germany; 20 Department of Pediatric Surgery and Pediatric Urology, Cnopfsche Kinderklinik-Klinik Hallerwiese, Nürnberg, Germany; 21 Department of Pediatric Surgery and Orthopedics, University Children’s Hospital Regensburg (KUNO) at the Hospital St. Hedwig of the Order of St. John, Regensburg, Germany; 22 Center of Pediatric Surgery Hannover, Hannover Medical School, Hannover, Germany; 23 Department of Pediatric Surgery, Evangelisches Krankenhaus Oberhausen, Oberhausen, Germany; 24 Department of Pediatric Surgery and Pediatric Urology, Goethe University Frankfurt, Frankfurt, Germany; 25 Department of Pediatrics, University of Zielona Góra, Zielona Góra, Poland; 26 Institute of Cardiovascular Regeneration, Center for Molecular Medicine, University of Frankfurt, Frankfurt am Main, Germany; 27 Department of Child and Adolescent Psychiatry, University Medical Center of the Johannes Gutenberg University Mainz, Mainz, Germany; 28 Faculty of Health, School of Medicine, University of Witten/Herdecke, Witten, Germany; 29 Division of Neonatology and Pediatric Intensive Care, Department of Pediatrics and Adolescent Medicine, Friedrich-Alexander University Nürnberg-Erlangen, Erlangen, Germany; 30 Department of Pediatrics and Adolescent Medicine, Friedrich-Alexander University Nürnberg-Erlangen, Erlangen, Germany; 31 Research Center On Rare Kidney Diseases (RECORD), University Hospital Erlangen, 91054 Erlangen, Germany

**Keywords:** Medical research, Pathogenesis, Risk factors

## Abstract

Anorectal malformations (ARM) represent a spectrum of rare malformations originating from a perturbated development of the embryonic hindgut. Approximately 60% occur as a part of a defined genetic syndrome or within the spectrum of additional congenital anomalies. Rare copy number variations (CNVs) have been associated with both syndromic and non-syndromic forms. The present study represents the largest study to date to explore the contribution of CNVs to the expression of ARMs. SNP-array-based molecular karyotyping was applied in 450 individuals with ARM and 4392 healthy controls. CNVs were identified from raw intensity data using PennCNV. Overlapping CNVs between cases and controls were discarded. Remaining CNVs were filtered using a stringent filter algorithm of nine filter steps. Prioritized CNVs were confirmed using qPCR. Filtering prioritized and qPCR confirmed four microscopic chromosomal anomalies and nine submicroscopic CNVs comprising seven microdeletions (del2p13.2, del4p16.2, del7q31.33, del9p24.1, del16q12.1, del18q32, del22q11.21) and two microduplications (dup2p13.2, dup17q12) in 14 individuals (12 singletons and one affected sib-pair). Within these CNVs, based on their embryonic expression data and function, we suggest *FOXK2*, *LPP*, and *SALL3* as putative candidate genes. Overall, our CNV analysis identified putative microscopic and submicroscopic chromosomal rearrangements in 3% of cases. Functional characterization and re-sequencing of suggested candidate genes is warranted.

## Introduction

Anorectal malformations (ARM) represent a spectrum of rare malformations originating from a perturbated development of the embryonic hindgut. ARM present with a male to female ratio of 1.7, the overall birth prevalence has been estimated with 1 in 2500–3000 live births [1]. Approximately 60% occur as a part of a defined genetic syndrome or within the spectrum of additional congenital anomalies, the remaining 40% present isolated non-syndromic [2]. The current knowledge of the underlying molecular mechanisms of isolated non-syndromic ARM is still limited. Based on observations such as reduced reproduction rate one can assume a significant fraction of underlying de novo events. The experience in medical genetics suggests that such mutational events will comprise genomic alterations of different size ranging from small changes affecting single nucleotides to large alterations resulting in losses or gains of several thousand to millions of base-pairs. To test this hypothesis, we previously, between 2011 and 2017, systematically employed array-based molecular karyotyping in altogether 224 individuals with syndromic and non-syndromic ARM [3–10]. In total, we identified a pathogenic de novo CNV in 5 % of cases. 11 cases presented with syndromic ARM according to Rasmussen et al. [11]. Accordingly, Wong et al. using molecular karyotyping in 363 Han Chinese with sporadic ARM and 4006 controls found an enrichment of rare long duplications among individuals with syndromic ARM [12]. The aims of our present study were (i) to explore the overall contribution of CNVs to the expression of ARMs, (ii) to identify novel disease-causing CNVs, and (iii) to identify novel candidate genes.

## Materials (subjects) and methods

### Cases and controls

Affected individuals and their families were recruited within the framework of the German “Network for Congenital Uro-REctal malformations” (www.cure-net.de). All experiments involving human materials adhered to the recommendations of the World Medical Association, as stated in the revised Declaration of Helsinki from Seoul 2008. The proposed project has been approved by the Ethics Commission of the University of Bonn. The study sample encompassed 450 individuals with ARM. A total of 212 individuals presented with a syndromic, 238 with a non-syndromic form. Furthermore, we included 4392 healthy controls [13]. For 351 affected individuals, the DNA of both parents was available, for 57 affected individuals, only maternal, and for 5 affected individuals, only paternal DNA was available. For 37 affected individuals no parental sample was available.

### DNA isolation and array-based molecular karyotyping

Genomic DNA of patients and their parents was isolated from the blood samples by using the Chemagic Magnetic Separation Module I (Chemagen, Baesweiler, Germany), or from saliva samples using the Oragene DNA Kit (DNA Genotek Inc., ON, Canada). Cases and Controls were genotyped using the single nucleotide polymorphism (SNP)-based array “Infinium Global Screening Array-24 v2.0”. This array contains >600.000 SNPs that are evenly distributed across the genome. All chromosomal positions are given in human genome build hg19 (GRCh37 Genome Reference Consortium Human Build 37 (GRCh37)).

### CNV filtering

CNV calling was accomplished using PennCNV. The algorithm of PennCNV uses a Hidden-Markov model to estimate putative CNVs from log R ratio (normalized intensity data) and B allele frequency (allele frequency data) of each SNP. Quality criteria used to exclude individuals due to failed genotyping were (I) a gender mismatch between assumed and called gender; (II) a call rate <98%; (III) a number of CNVs per affected individual that exceeded double of the standard deviation of the mean number of CNVs called per individual. Subsequent criteria to exclude single CNVs were (IV) maximum log Bayes factor below 30; and (V) if the CNV comprised less than 3 consecutive called SNPs. These steps were followed by filtering of the remaining CNVs for prioritization of potential disease-causing CNVs. Filter steps included (VI) comparison of CNVs in cases and inhouse controls. All CNVs identified in cases that were overlapped by one or more CNV form controls were not further considered potential disease causing. Beforehand, all 4394 inhouse controls had been quality checked accordingly using criteria (I)–(V). For the remaining CNVs the information for each CNV from PennCNV was combined with the information from the annotation and ranking tool AnnotSV, and filtered out in a manual filter step (VII) [14]. Here, CNVs comprising deletions were discarded when overlapping with deletion CNVs annotated in the Database of Genomic Variants (DGV) and CNVs comprising duplications were discarded when overlapping with duplication CNVs annotated in the Database of Genomic Variants (DGV). Furthermore, (VIII) CNVs that did not reside within coding or promotor regions were filtered out. (IX) Using GenomeStudio Genotyping Module v2.0 and the Illumina Genome Viewer version 2.0.4 the remaining CNVs were visually inspected. (X) Final priorization of the remaining CNV regions was performed by gathered information from publicly available databases: Gnomad (https://gnomad.broadinstitute.org), OMIM (https://www.omim.org), UCSC (https://genome.ucsc.edu), MGI (http://www.informatics.jax.org), and PubMed (https://pubmed.ncbi.nlm.nih.gov). Genome build hg19/GRCh37 was used for all data.

### CNV confirmation

To confirm all prioritized CNVs and to identify de novo events when parental DNA was available, quantitative polymerase chain reaction (qPCR) was carried out. SYBR Green (Applied Biosystems, Foster City, CA) was used for qPCR reactions. The evaluation of the copy numbers was performed using the comparative CT method (ΔΔCt method). Three housekeeping genes were used to compare relative copy counts (*CFTR, BNC1, RNA*). The primer sequences are shown in Supplementary Table 1.

## Results

### CNV analysis

After the application of quality filter step I (gender mismatch), two patients were excluded. Through step II (call rate <98%), 40 patients were discarded and after step III (exceeded double of standard deviation), 12 more patients were excluded. In the remaining 396 individuals with ARM, a total of 6316 CNVs, were called. Within the remaining 4066 controls, a total of 43,323 CNVs were called. Regarding the quality filter for each CNV, filter steps (IV) and (V) led to the exclusion of 5026 CNVs among ARM individuals and the exclusion of 32,075 CNVs among healthy control individuals. Comparison of frequencies in cases and controls (VI) excluded additional 887 CNVs. In this filter step, we identified three individuals with trisomy 21 (not further mentioned). Application of filter steps VII and VIII filtered out 117 microdeletions and 35 microduplications. Filter step IX yielded a final count of 82 microdeletions and 49 microduplications. According to the annotation of the respective CNVs in gnomAD (https://gnomad.broadinstitute.org), OMIM (https://www.omim.org), UCSC (https://genome.ucsc.edu), MGI (http://www.informatics.jax.org), and PubMed (https://pubmed.ncbi.nlm.nih.gov), we ultimately prioritized and qPCR confirmed four microscopic chromosomal anomalies and nine submicroscopic CNVs comprising seven microdeletions (del2p13.2, del4p16.2, del7q31.33, del9p24.1, del16q12.1, del18q32, del22q11.21) and two microduplications (dup2p13.2, dup17q12).

### Unbalanced chromosomal translocations

Two affected individuals carried unbalanced terminal translocations including 46,XX,der(11)t(11;16)(q24.2;q22.2) and 46,XX,der(22)t(22;9)(q11.21;pter) (Table 1).

**Table 1:** Unbalanced chromosomal translocations and microscopic chromosomal de novo duplications.

	Individual 1	Individual 2	Individual 3	Individual 4
CNV	46,XX,der(11)t(11;16) (q24.2;q22.2)	46,XX,der(22)t(22;9) (q11.21;p)	46,XY,dup(3) (q26.31-q29)	46,XY,dup(17) (q25.3-qter)
Type of ARM	Perineal fistula	Perineal fistula	–	Perineal fistula
Phenotypes				
Heart	–	–	–	–
CNS	Intraventricular hemorrhage of the newborn	–	–	–
Esophagus	–	–	–	–
Extremities	Bilateral clubfoot	–	–	–
Vertebral bodies	–	–	–	–
Hips	–	–	–	–
Kidney	Dysplastic pelvic kidney (left)	Kidney hypoplasia (left). Bilateral hydronephrosis	–	–
Urinary tract	–	Persistent urogenital sinus	–	–
Genital organs	–	–	–	–
Other	Bilateral atresia of submandibular gland. Left sided aplasia of thyroid. Choanal atresia. Hearing disability. Macular delocalization	Premature infant (36 + 0), small for gestation age (2090 g)	–	–

*ARM* anorectal malformation, *CNS* central nervous system.

Individual 1 presented with an ARM in form of a perineal fistula, congenital intraventricular hemorrhage with hydrocephalus, bilateral clubfoot, dysplastic pelvic kidney (left), bilateral atresia of submandibular gland, left sided aplasia of the thyroid gland, choanal atresia, hearing disability, and macular delocalization. Her chromosomal anomaly comprised an unbalanced translocation 46,XX,der(11)t(11;16)(q24.2;q22.2) with terminal deletion 11q24.2-qter. The first deleted SNP is located at chromosomal position chr11:127.078.525 and the last deleted SNP at chr11:134.923.456. Deletion of 11q23.3-qter has been associated with Jacobsen syndrome (JBS, MIM #147791). JBS is a contiguous gene deletion syndrome involving terminal chromosome 11q. Key phenotypic features are pre- and postnatal growth retardation, intellectual disability (ID), characteristic facial dysmorphism, thrombo- or pancytopenia. Further clinical features are congenital heart defects (CHD), congenital anomalies of the kidneys and urinary tract (CAKUT), gastrointestinal tract, genitalia, central nervous system (CNS) anomalies and/or skeleton. Previously, Mattina et al. reported gastrointestinal tract malformations in 18–25% of individuals with JBS including pyloric stenosis, ARM, and less frequently, duodenal atresia, annular pancreas, or gut malrotation [15]. Besides terminal deletion 11q24.2-qter Individual 1 carried also an 18 Mb duplication of chromosomal region 16q22.2-qter. The first duplicated SNP is located at chromosomal position chr16:71.956.505, the last duplicated SNP at chromosomal position chr16:90.161.959. Key features described for partial trisomy 16qter (ORPHA:96106) include low birth weight, failure to thrive, hypotonia, ID, CHD, limb anomalies and joint contractures, facial dysmorphism with a high prominent forehead, down slanting and small palpebral fissures, hypertelorism, periorbital edema, low-set and abnormal ears, prominent nose, micrognathia, CAKUT, genital anomalies, and ARM. Interestingly, several reports in the literature associated dup16pterqter with ARM [16].

Individual 2 presented with ARM in form of a perineal fistula, kidney hypoplasia (left), bilateral hydronephrosis, persistent urogenital sinus, and small for gestational age. Her chromosomal anomaly comprised an unbalanced translocation with a 2.5 Mb deletion of chromosomal region 22q11.21. The first deleted SNP was located at chromosomal position chr22:18.875.445 and the last deleted SNP was located at chromosomal position chr22:21.461.607. The 22q11.2 deletion syndrome is the most common microdeletion syndrome. It is not surprising that several cases of ARM have been described among patients with this deletion [16] (see below Individual 14). Individual 2 also carried a 38.8 Mb duplication of 9p. The first duplicated SNP was located at chromosomal position chr9:209.325, the last duplicated SNP was located at chromosomal position chr9:39.021.035. Common features of a 9p trisomy include ID, craniofacial dysmorphisms, skeletal alterations, CNS anomalies, CHD, and less common CAKUT. While ARM has not been associated with trisomy 9p, several reports in the literature mention ARM in association with mosaic or non-mosaic complete trisomy 9 [16].

### Microscopic chromosomal de novo duplications

Two affected individuals carried each a terminal duplication including 46,XXdup(3)(q26.31-q29) and 46,XXdup(17)(q25.3-qter) (Table 1).

**Fig. 1:**
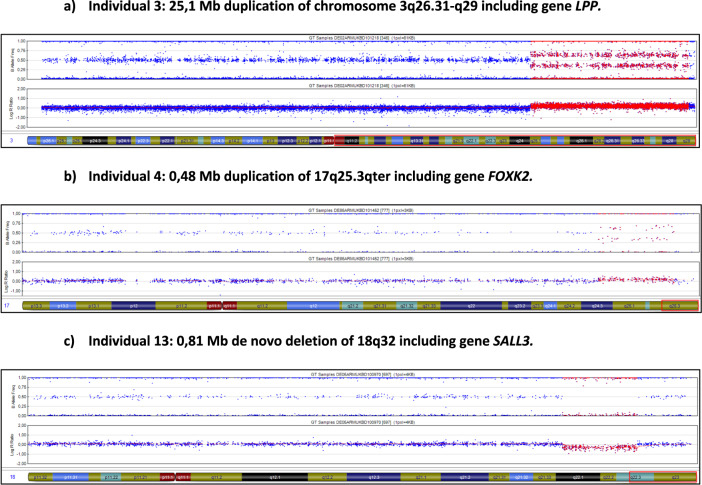
CNVs harboring putative ARM candidate genes.

Individual 3 presented with an isolated ARM. It is uncertain if he presented with additional minor stigmata. His chromosomal anomaly comprised a 25.1 Mb duplication of chromosome 3q26.31-q29 (Fig. 1a). The first duplicated SNP was located at chromosomal position chr3:171.615.261, the last at chromosomal position chr3:196.805.528. Common features of 3q duplication syndrome are a hirsutism, synophrys, broad nasal root, anteverted nares, downturned corners of the mouth, malformed ears, CHD, CAKUT and genital anomalies, ID, and growth retardation. To the best of our knowledge, this is the first report of duplication 3q26.31-q29 in an individual with ARM.

Individual 4 presented with an isolated ARM in form of a perineal fistula. He did not present with any other congenital anomaly or stigmata. His chromosomal anomaly comprised an 0.48 Mb duplication of 17q25.3-qter (Fig. 1b). The first duplicated SNP was located at chromosomal position chr17:80.545.076, the last at chromosomal position chr17:81.033.874. To the best of our knowledge, this is the first report of duplication of 17q25.3-qter in an individual with ARM.

### Rare inherited CNVs and CNVs of unknown inheritance

Six affected individuals carried very rare inherited submicroscopic CNVs (Table 2). All of these CNVs were absent in 4066 healthy inhouse controls (CNV frequency <0.0003) and DGV.

**Table 2:** Rare inherited CNVs and CNVs of unknown inheritance.

Individuals	Inherited CNVs	First and last deleted SNP	Size	Type	Genes within CNV
Individual 5	del2p13.2	Chr2:72.623.204-72.939.279	316,076 bp	Deletion	*EXOC6B*
Individuals 6 and 7	dup2p13.2	Chr2:72.882.934-73.015.587	132,654 bp	Duplication	*EXOC6B*
Individual 8	del4p16.2	Chr4:5.162.593-5.427.691	265,099 bp	Deletion	*STK32B*
Individual 9	del7q31.33	Chr7:126.392.369-126.439.453	47,085 bp	Deletion	*GRM8*
Individual 10	dup17q12	Chr17:33.786.176-33.968.102	181,927 bp	Duplication	*AP2B1, LINC02001, LOC107985033, PEX12, SLFN12L, SLFN14, SNORD7*
*CNV of unknown inheritance*					
Individual 11	del9p24.1	Chr9:4.973.080-5.000.904	27,825 bp	Deletion	*JAK2*

Individual 10 presented with ARM in form of a perineal fistula. He carried a 181.925 bp maternally inherited duplication of chromosomal region dup17q1.

Individual 5 presented with an isolated ARM in form of a recto-vestibular fistula. Besides her ARM, she had no co-occurring anomalies. She carried a maternally inherited 316.074 bp deletion of chromosomal region 2p13.2. The first deleted SNP was located on chromosomal position Chr2:72.623.204, the last at chromosomal position Chr2:72.939.279. Within this deletion resides the developmental gene *EXOC6B*. *EXOC6B* has been associated with autosomal-recessive inherited spondyloepimetaphyseal dysplasia with joint laxity, type 3 (MIM #618395).

Individuals 6 and 7 are brothers who both presented with ARM in form of a perineal fistula. In addition, Individual 6 presented with a testicular appendage and right-sided deafness. His brother presented with additional epispadias. Both carried a maternally inherited 132,652 bp duplication of chromosomal region 2p13.2. The first duplicated SNP was located at chromosomal position Chr2:72.882.934, the last at chromosomal position Chr2:73.015.587. Within this duplication resides the above-mentioned developmental gene *EXOC6B*. While the above *EXOC6B*-associated spondyloepimetaphyseal dysplasia with joint laxity, type 3 does not present with any visceral organ affection, the findings in individual 5 and in the two brothers 6 and 7 suggests a possible involvement of *EXOC6B* or the respective genomic region in ARM formation.

Individual 8 presented with ARM in form of a rectoprostatic fistula, aganglionosis of the Meissner’s plexus (submucosa, M. Hirschsprung) and craniofacial Goldenhar syndrome. He carried a paternally inherited 265.097 bp deletion of chromosomal region 4p16.2. The first deleted SNP was located at chromosomal position Chr4:5.162.593, the last SNP at chromosomal position Chr4:5.427.691. Within this deletion resides the developmental gene *STK32B*, which has been associated with Ellis-van Creveld syndrome, an autosomal-recessive skeletal dysplasia with co-occurring genital anomalies, e.g., hypospadias.

Individual 9 presented with an unclassified ARM as part of her VATER/VACTERL association, with further phenotypic data missing. She carried a paternally inherited 47,083 bp deletion of chromosomal region 7q31.33. The first deleted SNP was located at chromosomal position Chr7:126.392.369, the last at chromosomal position Chr7:126.439.453. Within this deletion resides the developmental gene *GRM8*, so far associated with any human disease.

Individual 10 presented with ARM in form of a perineal fistula. He carried a 181.925 bp maternally inherited duplication of chromosomal region 17q1. The first duplicated SNP was located at chromosomal position Chr17:33.786.176, the last duplication at chromosomal position Chr17:33.968.102. Within this duplication reside five coding genes comprising *AP2B*, *PEX12*, *SLFN12L*, *SLFN14*, and *SNORD7* and two long non-coding RNAs LINC02001 and LOC107985033. Of the coding genes, *PEX12* has been associated with the autosomal-recessive Peroxisome biogenesis disorder 3A (Zellweger, MIM #614859). *SLFN14* has been associated with the autosomal-dominant “Bleeding disorder, platelet-type, 20 (MIM #616913)”.

Individual 11 presented with ARM in form of a recto-vesical fistula as part of his VATER/VACTERL association. He presented with additional CHD, right-sided kidney agenesis, and cryptorchidism. His chromosomal anomaly comprised a 27.823 bp deletion of chromosomal region 9q24.1 (Table 2). The first deleted SNP was located on chromosomal position Chr9:49.73.080, the last at chromosomal position Chr9:5.000.904. Here, we had no parental samples available for testing and can therefore not conclude, if the CNV has occurred de novo or has been transmitted from a healthy parent. Within the deletion resides the developmental gene *JAK2*, a gene that has been associated with broad spectrum of human hematologic malignancies (MIM *147796) but not with congenital visceral anomalies.

### Rare submicroscopic de novo CNVs

In total we detected and confirmed three submicroscopic de novo CNVs (Table 3).

**Table 3:** Patients with de novo CNVs.

	Individual 12	Individual 13	Individual 14
CNV	del16q12.1	del18q32	del22q11.21
Type of ARM	Perineal fistula	Rectoprostatic fistula	Perineal fistula
Phenotypes			
Heart			PDA, VSD, pulmonary valve stenosis
CNS	–	–	–
Esophagus	–	–	–
Extremities	–	–	–
Vertebral bodies	–	–	–
Hips	–	–	–
Kidney	–	Duplex kidney with megaureter	–
Urinary tract	–	–	–
Genital organs	–	Maldescensus testis (right)	–
Other	Auricular hypoplasia (right)	–	Luxation of the arytenoid cartilages, Abdominal wall hernia

*ARM* anorectal malformation, *CNS* central nervous system, *PDA* patent ductus arteriosus, *VSD* ventricular septal defect, *NA* not available.

Individual 12 presented with an ARM in form of a perineal fistula and right-sided auricular hypoplasia. She carried a 1.38 Mb de novo deletion of chromosomal region 16q12.1. The first deleted SNP was located at chromosomal position chr16:50.135.837, the last at chromosomal position chr16:51.522.044. Within the deleted region reside nine coding genes including *ADCY7*, *HEATR3*, *PAPD5*, *BRD7*, *CYLD*, *NKD1*, *NOD2*, *SNX20*, and *SALL1*. Furthermore, the deletion comprised two microRNAs (MIR6771 and MIR3181) and four long non-coding RNAs (LINC02127, LINC02168, LINC02178, LOC101927272).

Individual 13 presented with ARM in form of a rectoprostatic fistula, duplicated kidney with megaureter, and right-sided maldescensus testis. He carried a 0.81 Mb de novo deletion of chromosomal region 18q32 (Fig. 1c). The first deleted SNP was located at chromosomal position chr18:76.501.085, the last at chromosomal position 18:77.373.296. Within the deletion reside three coding genes comprising *SALL3*, *ATP9B*, and *NFATC1*.

Individual 14 presented with an ARM in form of a perineal fistula. In addition, she presented with patent ductus arteriosus, ventricular septal defect and pulmonary valve stenosis. Furthermore, she had luxation of the arytenoid cartilages and an abdominal wall hernia. Her chromosomal anomaly comprised an isolated 2.58 Mb deletion of 22q11.21 (see above Individual 2). The first deleted SNP was located on chromosomal position Chr22:18.875.445, the last at chromosomal position Chr22:21.461.607. DECIPHER database (DatabasE of genomiC varIation and Phenotype in Humans using Ensembl Resources) [17] lists 1267 CNVs of chromosomal region 22q11.2. These comprise 667 heterozygous deletions. Within these 667 deletions are 14 deletions that have been described in association with ARM. The smallest region of overlap of all these deletions comprises 1.39 Mb. Here, further genetic studies are warranted to prioritize a possible ARM associated human disease-gene.

## Discussion

Congenital ARM represent a rare but severe spectrum of birth defects of the hindgut, with life-long impairments. The current knowledge of the underlying molecular mechanisms is still limited.

The primary aim of our present study was to identify novel highly penetrant structural genomic variants at the size of CNVs, and to describe in-depth the overall contribution of highly penetrant CNVs to the expression of the ARM spectrum. Among 450 ARM individuals, we identified three individuals with trisomy 21, two unbalanced chromosomal translocations, and two microscopic large de novo duplications (Table 1). Furthermore, we detected and confirmed five very rare, putatively novel submicroscopic CNVs in six affected individuals (four independent individuals and one sib-pair) (Table 2). None of these CNVs were found in any of our 4,066 healthy inhouse controls (CNV frequency <0.0003) nor in DGV. To the best of our knowledge, none of these inherited CNVs have been previously associated with human ARM phenotypes or other human diseases (PubMed search February 2022). Furthermore, we detected and confirmed three submicroscopic de novo CNVs in three independent ARM individuals. Our results confirm our own previous observations in which about 5% of affected individuals were found to carry putative disease-causing CNVs.

All large microscopic chromosomal rearrangements detected here comprise previously syndromic ARM associated regions. Within the rare inherited CNVs, we were unable to identify any candidate gene that would have stood out due to its function or association with known human ARM related disease. However, our findings in Individual 5 and the brothers Individual 6 and 7 suggest a possible dosage effect of *EXOC6B* to be involved in human ARM formation. Biallelic recessive variants in *EXOC6B* have been associated with spondyloepimetaphyseal dysplasia with joint laxity, type 3, which does not present with visceral organ affection. However, according to Mouse Genome Database (MGD) *Exoc6b* shows strong expression in the mouse visceral organ system during embryonic development from embryonic day (ED) 10.5–18, including the critical embryonic timeframe for ARM organ formation [18]. Furthermore, we detected a heterozygous deletion in Individual 8 comprising *STK32B*, which has been associated with Ellis-van Creveld syndrome, an autosomal-recessive skeletal dysplasia with co-occurring genital anomalies, e.g., hypospadias. Hence, *STK32B* dosage effects might as well be implicated in ARM formation as assumed for *EXOC6B*, especially since we did not explore the respective trans alleles for disease-causing variants on the level of single base-pairs.

In five individuals we detected microscopic and submicroscopic chromosomal de novo rearrangements in which we prioritized three putative candidate genes. In Individual 12 we detected a 1.38 Mb de novo deletion of chromosomal region 16q12.1 (Table 3). Within the deleted region reside nine coding genes including *ADCY7*, *HEATR3*, *PAPD5*, *BRD7*, *CYLD*, *NKD1*, *NOD2*, *SNX20*, and *SALL1*. Previously, heterozygous deletions spanning *SALL1* have been associated with Townes–Brocks syndrome (MIM # 107480) [19]. Key phenotypic features of Townes–Brocks syndrome include microcephaly, auricular anomalies such as microtia, CHD, ARM, hypospadias, CAKUT, skeletal anomalies of the hands and feet. Hence, the present genetic finding and the individual’s phenotype suggest the diagnosis of Townes–Brocks syndrome in Individual 12.

In Individual 13 we detected and confirmed a 0.81 Mb de novo deletion of chromosomal region 18q32 (Table 3 and Fig. 1c). Within the deletion reside three coding genes comprising *SALL3*, *ATP9B*, and *NFATC1*. Previously, the two developmental genes *SALL1* and *SALL4* have been associated with syndromic ARM [19, 20]. Furthermore, DECIPHER database [17] lists 47 individuals with heterozygous de novo losses of the *SALL3* comprising genomic region. Two individuals presented with vaginal atresia, five with hypospadias, three with renal agenesis, and two with vesicoureteral reflux. Moreover, according to MAMEP database (http://mamep.molgen.mpg.de/index.php), *Sall3* is expressed in the early mouse embryo (Theiler stage, TS15) in distinct structures. The highest expression is detected in the neural tube along the complete body axis and in the caudal end mesenchyme. Additional expression domains are detected in the developing limb buds and the brain area. In younger TS12 embryos, weaker staining is also detected in the endoderm areas, from which the hindgut will develop [21]. Together with these previous findings and the here observed findings in Individual 13, we suggest a dosage effect of *SALL3* to be involved in the formation of human ARM or uro-rectal malformations in a broader spectrum. In Individual 3 we detected a 25.1 Mb duplication of chromosome 3q26.31-q29 (Fig. 1a). Within this large duplication resides among other genes the developmental gene *LPP*. Genetic variants of different size in *LPP* have been previously associated with the VATER/VACTERL association [22–25]. The acronym VATER/VACTERL association (OMIM #192350) refers to the non-random co-occurrence of the following component features (CFs): vertebral defects (V), anorectal malformations (ARM) (A), cardiac defects (C), tracheoesophageal fistula with or without esophageal atresia (TE), renal malformations (R), and limb defects (L) [24]. Individuals may present with additional anomalies; however, the clinical diagnosis requires the presence of at least three CFs [22]. These previously associated dosage effects of *LPP* include an approximate 451,000 bp heterozygous loss spanning nucleotides 189,395,885–189,951,376 on chromosome 3q (nucleotide position based on hg18) comprising only *LPP* gene [22]. According to MAMEP database *Lpp* is expressed widespread in TS15 embryos with distinct expression domains in the developing somites and the mesenchyme [21]. Additional expression domains are detected in the gut and in older TS17 embryos expression can be also detected in the hindgut area [21]. Together, we suggest *LPP* as a putative ARM candidate gene. In Individual 4 we detected a 0.48 Mb duplication of 17q25.3-qter (Fig. 1b). Within this duplicated region resides the developmental gene *FOXK2*. Very recently, the FOX-gene-cluster on human chromosome 16q24 has been associated with multifactorial esophageal atresia, the most common congenital intestinal atresia of the upper intestinal tract [26]. Furthermore, *Foxk2* has a widespread expression pattern in the TS15 stage mouse embryo with distinct expression domains in the branchial arches, developing limbs and the epithelial structure of the forebrain and midbrain [27]. Moreover, *Foxk2* is strongly expressed in almost all developing urinary tract structures at mouse embryonic day 15.5 with distinct expression patterns in mouse embryonic kidney [28]. Taken together, our findings and previous observations suggest dosage effects of *FOXK2* to be implicated in human ARM formation. Our focus on CNVs comprising coding regions limited our current and previous analysis. Thereby we might have missed deep intronic small CNVs affecting only regulatory regions such as TATA boxes or TAD boundaries, possibly implicated in the formation of human ARM [29]. An additional limitation of our study was the method we carried out for CNV calling. For molecular karyotyping we employed an SNP-based array containing >600,000 SNPs distributed across the genome, followed by the estimation of the CNV-positions through PennCNV based on gains or losses of the SNPs. As a result, the precise CNV breakpoints remain unknown. We therefore cannot determine exactly, if patients with the apparent same CNV such as patients 5, 6 and 7 share the exact same breakpoints.

Further studies on our generated SNP-based array datasets, which were clearly beyond the scope of our present study might elucidate the complete contribution of CNVs to the formation of human ARM and explain larger proportions of its unknown genetic background.

## Conclusions

This large scale CNV analysis with a focus on high penetrant chromosomal rearrangements in 450 individuals with ARM identified putative disease-causing microscopic and submicroscopic chromosomal rearrangements in 3% of cases. Within these CNVs reside putative candidate genes *FOXK2*, *LPP*, and *SALL3*. The confirmation of the putative candidate genes as ARM disease-genes will lead to new diagnostic possibilities, provide families and clinicians with greater knowledge of the causes of the disorder, and allow a precise estimation of a recurrence risk.

## Supplementary information

Supplementary Figure 1a

Supplementary Figure 1b

Supplementary Figure 1c

Supplementary Table 1

## Data Availability

CNV data that support the findings of this study have been deposited in “ClinVar-Database” [“https://www.ncbi.nlm.nih.gov/clinvar/”] with the accession codes SCV002576529–SCV002576543.
