# Supplementary figures and images for: Genome-wide identification of disease-causing copy number variations in 450 individuals with anorectal malformations
Source: Eur J Hum Genet. 2022 Nov 1;31(1):105–11. doi: 10.1038/s41431-022-01216-5 (PMC9822900; doi:10.1038/s41431-022-01216-5)

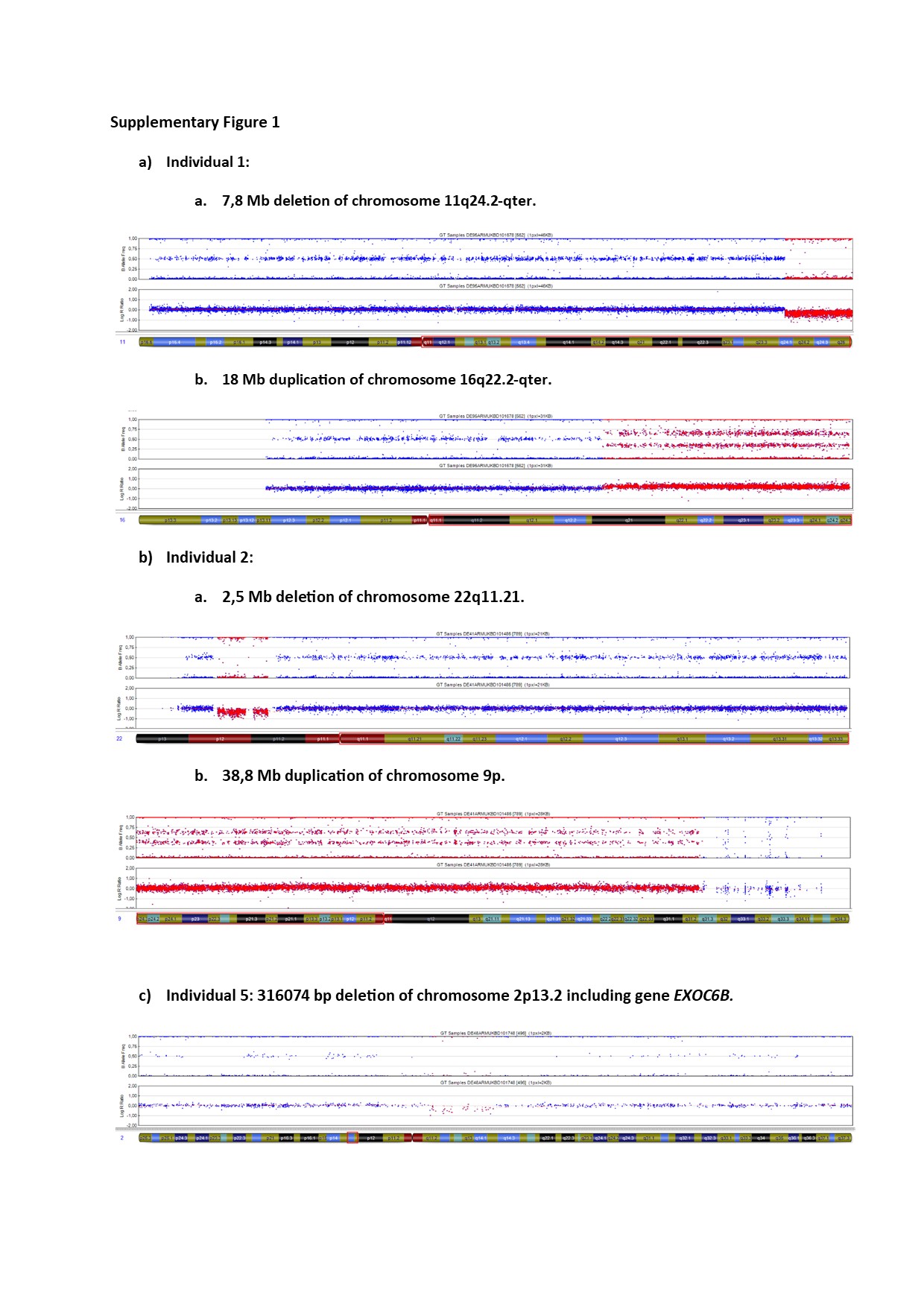

Supplement: Supplementary file 1 — Supplementary Figure 1a [file 41431_2022_1216_MOESM1_ESM.jpg]

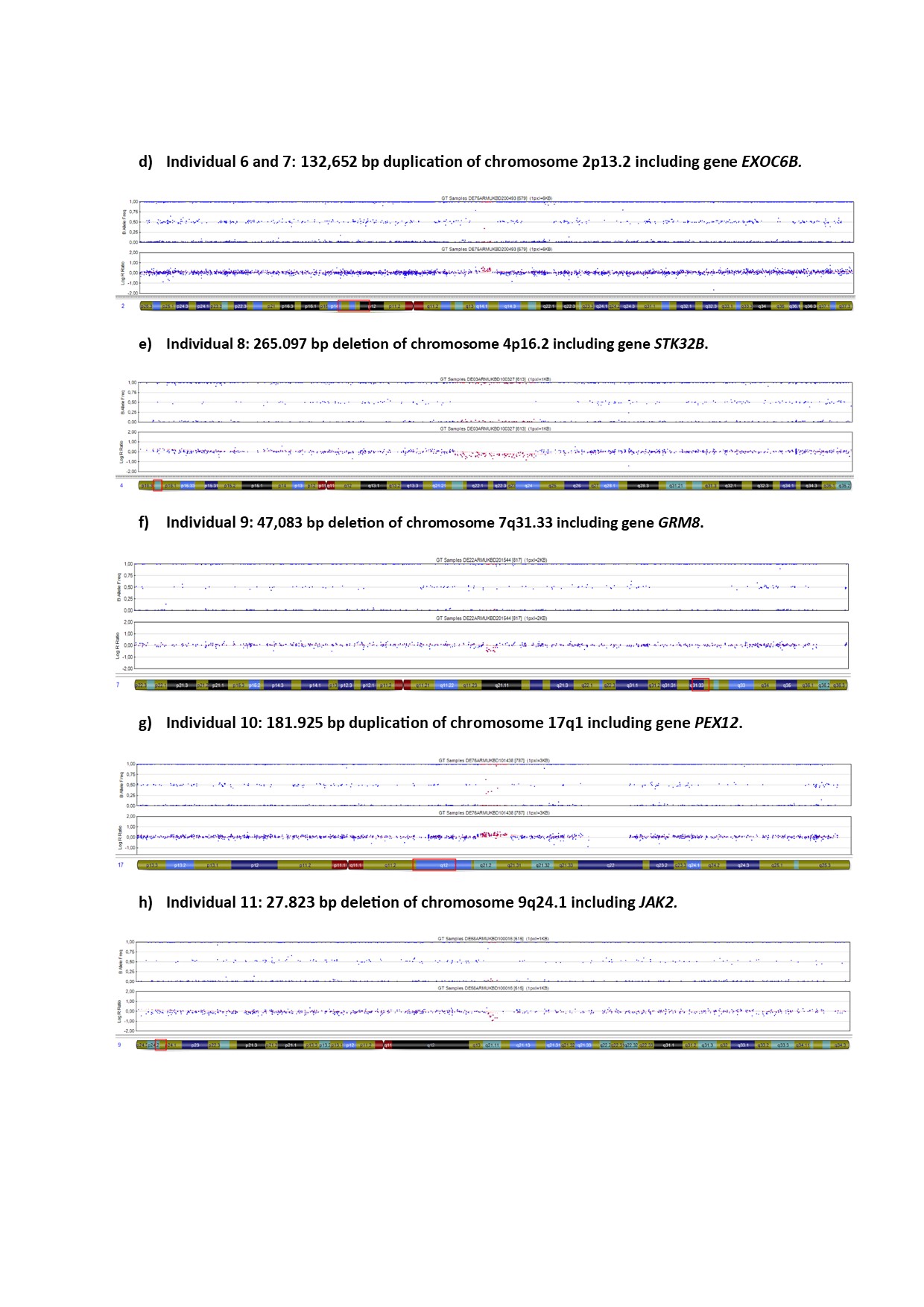

Supplement: Supplementary file 2 — Supplementary Figure 1b [file 41431_2022_1216_MOESM2_ESM.jpg]

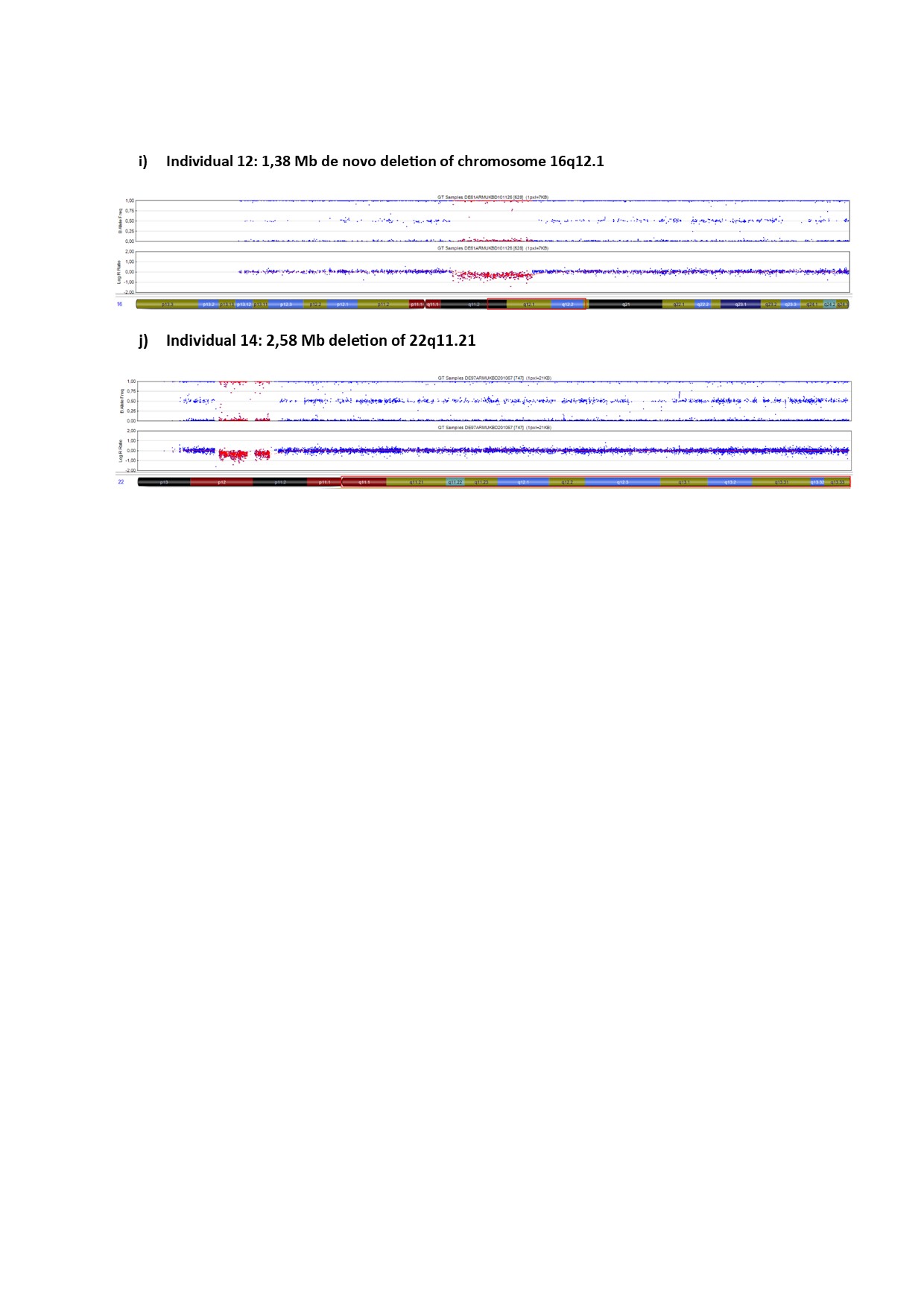

Supplement: Supplementary file 3 — Supplementary Figure 1c [file 41431_2022_1216_MOESM3_ESM.jpg]
