# Supplementary material for: Genome-wide identification of disease-causing copy number variations in 450 individuals with anorectal malformations
Source: Eur J Hum Genet. 2022 Nov 1;31(1):105–11. doi: 10.1038/s41431-022-01216-5 (PMC9822900; doi:10.1038/s41431-022-01216-5)
Supplement: Supplementary file 4 — Supplementary Table 1 [file 41431_2022_1216_MOESM4_ESM.docx]

**Supplementary Table 1**

**Primers used in qPCR.**

| **CNV** | **Primers** | **Sequence (5’-3’)** | **Position** | **Size** |
| --- | --- | --- | --- | --- |
| Del4p16.2 | Del4p16.2_1F | TCGCTCAGGTATGCATCGTG | chr4:5,170,018-5,170,037 | 117 bp |
|  | Del4p16.2_1R | GCAGCTCCCGGAAAACATTC | chr4:5,170,115-5,170,134 |  |
|  | Del4p16.2_2F | TGGGAGGTTGCTTTGGACAA | chr4:5,269,710-5,269,729 | 101 bp |
|  | Del4p16.2_2R | GCATTTTCAGTCCACAGGCTG | chr4:5,269,790-5,269,810 |  |
|  | Del4p16.2_3F | AGACTTCAACATAGCGACGGT | chr4:5,418,588-5,418,608 | 98 bp |
|  | Del4p16.2_3R | AAAGAAGGGGCCTGGAACAC | chr4:5,418,666-5,418,685 |  |
| Del4p16.1 | Del4p16.1_1F | CCCCTGCCACATCAAGAAGT | chr4:6,303,811-6,303,830 | 112 bp |
|  | Del4p16.1_1R | CTTGGTGACGTCGTCCTCCTCG | chr4:6,303,900-6,303,922 |  |
|  | Del4p16.1_2F | GCCAGCAGCGAGTTCAAGAGCGT | chr4:6,303,938-6,303,961 | 109 bp |
|  | Del4p16.1_2R | GCTCGAAGACAGGCCACTTG | chr4:6,303,916-6,303,935 |  |
|  | Del4p16.1_4F | AGATCCTCTGCAGCCACCTGGAG | chr4:6,303,582-6,303,604 | 116 bp |
|  | Del4p16.1_4R | GAACGGGAGCATGTTGATGG | chr4:6,303,678-6,303,697 |  |
| Del8q21.3 | Del8q21.3_1F | GACAAGGTGAGTGCATTCTTCTG | chr8:90,992,989-90,993,011 | 120 bp |
|  | Del8q21.3_1R | CCTTTGGTTGCATGCTCTTCTT | chr8:90,993,087-90,993,108 |  |
|  | Del8q21.3_2F | ACCACCAGCGTTATCATGAGT | chr8:90,994,325-90,994,345 | 116 bp |
|  | Del8q21.3_2R | CTTATGCCCCACAGCTCCTT | chr8:90,994,421-90,994,440 |  |
|  | Del8q21.3_3F | TGGCGCTCTCCATACTCTCT | chr8:90,996,082-90,996,101 | 104 bp |
|  | Del8q21.3_3R | GTGGGTGTGAAGGGTCTTGT | chr8:90,996,166-90,996,185 |  |
| Del13q31.3 | Del13q31.3_1F | GGTGTGCCTGAACAGAGCTA | chr13:93,391,514-93,391,533 | 97 bp |
|  | Del13q31.1_1R | TGCTCTTCATGGCTTTAGGTCA | chr13:93,391,589-93,391,610 |  |
|  | Del13q31.1_2F | TCACCTGTGCTTTCTTATTTGCC | chr13:93,518,505-93,518,527 | 118 bp |
|  | Del13q31.1_2R | CATCCTGCTCCTGTTGTGTCT | chr13:93,518,602-93,518,622 |  |
|  | Del13q31.1_3F | TCACCCTTCTGAGTTGTGGC | chr13:93,603,657-93,603,676 | 97 bp |
|  | Del13q31.1_3R | CTGTAGCAGGGCCTCTCTTG | chr13:93,603,734-93,603,753 |  |
| Del16q12.1 | Del16q12.1_1F | TTGTCCATCCGTTTGCCTGA | chr16:50,140,548-50,140,567 | 90 bp |
|  | Del16q12.1_1R | ACTGGGGATTGGGCCTTTTG | chr16:50,140,618-50,140,637 |  |
|  | Del16q12.1_2F | GGACCCTGCTCCTAATGCTC | chr16:50,655,472-50,655,491 | 93 bp |
|  | Del16q12.1_2R | TTCTTCTCATCTCCGCTGCC | chr16:50,655,545-50,655,564 |  |
|  | Del16q12.1_3F | TCCCTGGCTCTCTCTGTGTT | chr16:51,431,944-51,431,963 | 100 bp |
|  | Del16q12.1_3R | TGCCCACTGTTTCTGTCTCC | chr16:51,432,024-51,432,043 |  |
| Del18q32 | Del18q32_1F | CTGAGCTTCGTCTAGGGCAG | chr18:76,511,934-76,511,953 | 103 bp |
|  | Del18q32_1R | GGAGCAGTGAAAGCAACAGC | chr18:76,512,017-76,512,036 |  |
|  | Del18q32_2F | GCGTTTTCAGCGTGACAAGG | chr18:76,886,317-76,886,336 | 90 bp |
|  | Del18q32_2R | ACGCTAAGCAGGATTAAAGCATC | chr18:76,886,384-76,886,406 |  |
|  | Del18q32_3F | CAGCTAGCCGGTCTGTTTCT | chr18:77,358,914-77,358,933 | 109 bp |
|  | Del18q32_3R | GTGCGCTGGTTTATGATGGG | chr18:77,359,003-77,359,022 |  |
| Dup11q23.3 | Dup11q23.3_1F | CAGTGAGGCAGAAACAGATCCT | chr11:120,302,530-120,302,551 | 90 bp |
|  | Dup11q23.3_1R | TCTGCATTGTCACTACTGGGC | chr11:120,302,599-120,302,619 |  |
|  | Dup11q23.3_2F | TTCATAGAAGATGCCCCGCG | chr11:120,531,018-120,531,037 | 104 bp |
|  | Dup11q23.3_2R | GGCCACACTTACCGATCCTC | chr11:120,531,102-120,531,121 |  |
|  | Dup11q23.3_3F | TCCTTCTCTTCGCCTTGCAG | chr11:120,707,480-120,707,499 | 104 bp |
|  | Dup11q23.3_3R | CCAAGGAGGTGAGCTGTGAG | chr11:120,707,564-120,707,583 |  |
| Dup17q12 | Dup17q12_1F | TGTACATCAACACAGGCCCTT | chr17:33,806,660-33,806,680 | 99 bp |
|  | Dup17q12_1R | TTCTGCTGCACTGGAGTTCC | chr17:33,806,739-33,806,758 |  |
|  | Dup17q12_2F | AGCATCATGACCACCCACTG | chr17:33,884,036-33,884,055 | 98 bp |
|  | Dup17q12_2R | TTCTGTTGCGTGGTGTTTGC | chr17:33,884,114-33,884,133 |  |
|  | Dup17q12_3F | GTCACTTTGCTGTCTGGGGA | chr17:33,953,788-33,953,807 | 94 bp |
|  | Dup17q12_3R | ACACTTCGCTATTTCCCAACCT | chr17:33,953,860-33,953,881 |  |
| DupDel2p13.2 | DupDel2p13.2_1F | ACGAAGAAAGGCAATGAGGTCT | chr2:72,692,319-72,692,340 | 90 bp |
|  | DupDel2p13.2_1R | TTCCTACAGCTGGCAGACTATG | chr2:72,692,387-72,692,408 |  |
|  | DupDel2p13.2_2F | TGAAGTACCAAACGAGCCTGT | chr2:72,742,188-72,742,208 | 109 bp |
|  | DupDel2p13.2_2R | GGTCCAATTGTGTCAACCCATTT | chr2:72,742,274-72,742,296 |  |
|  | DupDel2p13.2_3F | ACACACAGACACCCCTTCAC | chr2:72,903,114-72,903,133 | 111 bp |
|  | DupDel2p13.2_3R | AGAGCCTGGTCTGTTCATGC | chr2:72,903,205-72,903,224 |  |
|  | DupDel2p13.2_4F | ACAGTTTCATAGTAAGACCTGCCA | chr2:72,958,476-72,958,499 | 93 bp |
|  | DupDel2p13.2_4R | CTTGGGGAGAAAGCGGGAAA | chr2:72,958,549-72,958,568 |  |
|  | DupDel2p13.2_5F | AGCTGCATTCATGTAACTTGGC | chr2:73,007,685-73,007,706 | 90 bp |
|  | DupDel2p13.2_5R | TTCTACTCCATGTCCTGTTGCC | chr2:73,007,753-73,007,774 |  |
| Del7q31.33 | Del7q31.33_1F | TGCAAAGACTTTTCAATCACGGT | chr7:126,393,995-126,394,017 | 96 bp |
|  | Del7q31.33_1R | TCATTATCCGGCGGTGGTTC | chr7:126,394,071-126,394,090 |  |
|  | Del7q31.33_2F | GCCCGAATATAACCAAGTAGCTCT | chr7:126,409,933-126,409,956 | 107 bp |
|  | Del7q31.33_2R | GCTTACGCCCTGCACAATATG | chr7:126,410,019-126,410,039 |  |
|  | Del7q31.33_3F | ACCTTCACTGAGTTAACATGGCA | chr7:126,436,526-126,436,548 | 100 bp |
|  | Del7q31.33_3R | TTCCTATGAGCCACTCCCCT | chr7:126,436,606-126,436,625 |  |
| Dup7q31.33 | Dup7q31.33_1F | CAGCCACCAAGATATACAGGGAA | chr7:125,947,591-125,947,613 | 102 bp |
|  | Dup7q31.33_1R | ATTCGTAGGTTCTTGGCCCC | chr7:125,947,673-125,947,692 |  |
|  | Dup7q31.33_2F | GGATGGCAGATACCTCGCAA | chr7:126,082,978-126,082,997 | 116 bp |
|  | Dup7q31.33_2R | AGCATATTCATCAGCACCGTGA | chr7:126,083,072-126,083,093 |  |
|  | Dup7q31.33_3F | GTCCGCTGCTCTCCATAGTC | chr7:126,173,247-126,173,266 | 93 bp |
|  | Dup7q31.33_3R | CTTCAGCCTCATCTCCGTCC | chr7:126,173,320-126,173,339 |  |
| Housekeeping genes | CFTR_F | GGAGATGCTCCTGTCTCCTG | chr7:117,232,238-117,232,257 | 138 bp |
|  | CFTR_R | GGGAGTCTTTTGCACAATGG | chr7:117,232,356-117,232,375 |  |
|  | BNC1_F | TCAGTGCTTTGTCCAACAGG | chr15:83,931,933-83,931,952 | 125 bp |
|  | BNC1_R | GCAGATGTCACACTGGAAGC | chr15:83,931,828-83,931,847 |  |
|  | RNA_F | CTGCCATGATCACCTCACAC | chr10:15,145,732-15,145,751 | 128 bp |
|  | RNA_R | GAACGCCAAGGCTAGAACAC | chr10:15,145,840-15,145,859 |  |
